# Canine Multidrug-Resistant *Pseudomonas aeruginosa* Cases Linked to Human Artificial Tears–Related Outbreak

**DOI:** 10.3201/eid3012.240085

**Published:** 2024-12

**Authors:** Emma R. Price, Darby McDermott, Adrienne Sherman, Lakisha Kelley, Jason Mehr, Rebecca Greeley, Stephen D. Cole

**Affiliations:** Centers for Disease Control and Prevention, Atlanta, Georgia, USA (E.R. Price), New Jersey Department of Health, Trenton, New Jersey, USA (E.R. Price, D. McDermott, A. Sherman, L. Kelley, J. Mehr, R. Greeley), University of Pennsylvania School of Veterinary Medicine, Philadelphia, Pennsylvania, USA (S.D. Cole)

**Keywords:** Pseudomonas aeruginosa, bacteria, carbapenems, antimicrobial resistance, microbial, dogs, pets, veterinary medicine, One Health, disease outbreaks, New Jersey, United States

## Abstract

We report 2 canine cases of carbapenemase-producing *Pseudomonas aeruginosa* within a United States veterinary hospital associated with a human outbreak linked to over-the-counter artificial tears. We investigated veterinary hospital transmission. Veterinary antimicrobial resistance surveillance and infection prevention and control enhancements are needed to reduce transmission of carbapenemase-producing organisms.

Carbapenem antimicrobial drugs are reserved for highly resistant gram-negative bacterial infections. Carbapenemase enzymes, which hydrolyze and inactivate carbapenems, are commonly encoded on mobile genetic elements that can spread among bacterial genera and species and amplify resistance. Therefore, carbapenemase-producing organisms (CPOs) are a major public health concern (*1*). Although less commonly documented compared with humans, CPOs have been identified in companion animals and suspected transmission reported between humans and animals (*2*–*4*).

In March and June 2023, New Jersey Department of Health (NJDOH) was notified of carbapenemase-producing *Pseudomonas aeruginosa* (CP-PsA) isolated from 2 separately owned pet dogs treated at the same New Jersey, USA, small animal specialty veterinary hospital. The isolates were closely genetically related to the multistate cluster of Verona integron‐mediated metallo‐β‐lactamase (VIM)–producing and Guiana‐extended spectrum‐β‐lactamase (GES)–producing carbapenem‐resistant *P. aeruginosa* (VIM‐GES‐CRPA) isolated from multiple human clinical cultures and associated with contaminated over-the-counter artificial tears products (*5*,*6*). The combination of VIM-80 and GES-9 in a single organism had not been identified in the United States before that outbreak. By May 2023, that outbreak was associated with 81 human cases and 4 deaths in 18 states; no other animal cases were reported.

NJDOH interviewed the dog owners, reviewed veterinary medical and hospital purchase records, and conducted an onsite infection prevention and control (IPC) assessment 1 month after the second case identification. The investigation was reviewed by Centers for Disease Control and Prevention (CDC) and conducted consistent with federal law and CDC policy. 

The first canine case was identified in March 2023 in a spayed female Labrador retriever 7 years of age that had a 3-month history of cough. VIM‐GES‐CRPA was isolated from a bronchoalveolar lavage specimen. The second canine case was identified in June 2023 in a neutered male cocker spaniel 6 years of age with a chronic history of otitis externa and keratoconjunctivitis sicca; VIM‐GES‐CP-PsA was isolated from the external ear canal along with methicillin-resistant *Staphylococcus pseudintermedius*. Clinical specimens were submitted to the clinical microbiology laboratory of the PennVet Diagnostic Laboratory, University of Pennsylvania (Philadelphia, PA, USA), for culture and antimicrobial susceptibility testing (AST).

AST was performed using AST-GN98 cards on Vitek 2 (bioMérieux, https://www.biomerieux.com), according to manufacturer instructions. Isolates tested were resistant to aminoglycosides amikacin and gentamicin, fluoroquinolones enrofloxacin and marbofloxacin, and ceftazidime. Isolate 13494-23 was resistant to imipenem. Although isolate 30793-23 was susceptible (MIC 2 μg/mL), ceftazidime resistance still prompted PCR by Carba-R (Cepheid, https://www.cepheid.com) for carbapenemase genes. Phenotypically susceptible isolates producing carbapenemases are a well-described phenomenon and prompt further investigation (*7*). Short-read whole-genome sequencing was performed using Nextera Library Prep chemistry and HiSeq 2500 (Illumina, https://www.illumina.com) platform and uploaded to National Center for Biotechnology Information (https://www.ncbi.nlm.nih.gov) prokaryotic genomic annotation pipeline for deposit in the pathogen detection database (*8*). The isolates were 2 and 5 single-nucleotide polymorphism differences from the closest related human isolate (Table)_._

**Table:** Clinical characteristics and genetic relatedness of *Pseudomonas aeruginosa* infections in canines during outbreak in humans linked to artificial tears*

Case no.	Sample collection month, 2023	Isolate location	Isolate no.	BioSample ID†	SNP difference from human outbreak strain
Dog 1	March	Bronchial alveolar lavage	13494-23	SAMN33902373	2
Dog 2	June	External ear canal	30793-23	SAMN35751022	5

*ID, identification; SNP, single nucleotide polymorphism. †National Center for Biotechnology Information (https://www.ncbi.nlm.nih.gov).

Neither dog owners nor household members reported outbreak-associated ophthalmic product exposures since March 2022, but the second dog had received different over-the-counter artificial tears. The veterinary hospital did not stock the outbreak-associated products. Both dogs had received recent antimicrobial drug treatment. The first dog lived with 3 other dogs; the second dog was the only household pet. No dogs, owners, or household members had travel history (domestic or international) or healthcare setting exposures. Epidemiologic links between the 2 canine cases included treatment in the veterinary facility’s surgical preparation and recovery areas for both dogs and ophthalmology department visits by either the affected dog or another animal in the same household. The NJDOH onsite visit identified IPC gaps in hand hygiene, personal protective equipment use, and equipment and environmental cleaning and disinfection. Surgical scrub and instrument sink drains, shared equipment, and ophthalmic product cultures did not grow the outbreak strain. NJDOH provided IPC resources and recommendations to the facility and owners.

CPO identification in dogs linked to a human outbreak but with an unknown transmission route necessitates consideration of the role of companion animals and veterinary hospitals in transmitting and acting as reservoirs for CPOs and underscores the need for veterinary public health action. To clarify veterinary-associated CPO transmission and enhance CPO identification, veterinarians should request diagnostic laboratories perform carbapenem susceptibility testing for gram-negative bacteria resistant to third-generation cephalosporins (e.g., ceftazidime), if clinical history suggests CPO infection risk, and, upon carbapenem-resistant organism identification, request resistance mechanism confirmation. CPO infection risk can include recent antimicrobial use, international travel, hospitalization, raw food diet, close contact with humans or animals carrying CPOs, or exposure to contaminated products (*9*,*10*). Veterinarians and pet owners are encouraged to maintain awareness of outbreaks in persons associated with products used in multiple species, such as through Food and Drug Administration medical product recall notifications. 

In conclusion, identifying CPOs in companion animals associated with a human outbreak serves as an urgent call to veterinarians to identify and prevent CPO transmission. Veterinarians should request carbapenem susceptibility testing when appropriate, veterinarians and pet owners should maintain awareness of CPO outbreaks, and veterinary hospitals should establish and implement IPC protocols. By following those recommendations, veterinarians can identify and prevent CPO transmission to protect animal and human health.
